# Medical Items

**Published:** 1916-12

**Authors:** 


### MEDICAL ITEMS

### A WIDELY USEFUL SOAP.

There are a number of so-called antiseptic soaps. Probably the most generally serviceable of these is Germicidal Soap, formula of Dr. Charles T. McCintock, which has been not inapty designated "the soap of a hundred uses"—a soap made from pure vegetable oils and containing the powerful antiseptic mercuric iodide. As indicative of the germicidal power of ths soap it may be said that a solution of it containing one part of mercuric iodide in five thousand parts of diluent will destroy pus organisms in less than five minutes. It is undoubtedly the most available antiseptic for the general practitioner. There are no solutions to carry. The soap is always ready for use. It does not stain linen or tarnish polished instruments.

Some of the uses to which Gerimcidal Soap is adopted are these: To prepare antiseptic solutions; to sterilize the hands, instruments and site of operation; to cleanse wounds, ulcers, etc.; to lubricate sounds, specula and catheters; to destroy infecting organisms in skin diseases; to disinfect surface lesion; to control itching in skin affections; to make solutions for the vaginal douche; to destroy offensive odors; to cleanse the hair and scalp and remove and prevent dandruff; to disinfect vessels, utensils, etc.; to wash and sterilize bed-linen used in the sick-room. It is apparent from the foregoing that the soap is at once an antiseptic, disinfectant, deodorant, sterilizer, lubricant and cleanser.

As most physicians probably know, Germicidal Soap, McClintock, is manufactured by Parke, Davis & Co. It is supplied in two strengths, containing, respectively, one per cent. and two per cent. of mercuric iodide. It is well to specify "P. D. & Co." when ordering from the druggist.

---

### HARD DRY FECES.

"Interol" is suggested as a means of overcoming this difficulty—and a hard, dry fecal mass is indeed a difficulty—because "Interol" has several points in its favor.

In the first place, it becomes part of the intestinal contents as they emerge from the cecum into the colon. It is thus mixed with them, and covering them. Under its influence, feces can not become hard and dry. The colon may absorb all the water it wants, but "Interol" remains with the mass all through its colonic and rectal journey, finally lubricating it past the sphincter ani during the defication act.

By so doing, straining at stool, which is an invariable accompaniment of hard dry feces, no longer is a necessity, and herein lies the value of "Interol," not only as a fecal softener, and lubricant, but as a prophylactic measure in the prevention of the many physical sequelae of straining at stool, including hernia, hemorrhoids and prolapse (rectal and uterine.)*

*Four-page circular on "Hard Dry Feces" sent on request. Also four-page circular on "Straining at Stool." Or Interol-lubrication booklet. Van Horn and Sawtell, _5-_7 East 40th Street,t N. Y.

### Obstipation Following Operation.

There are many theoretical reasons why INTEROL should be of value to the post-operatively constipated patient. But the best reason is that it IS of value.

And the most gratifying about it is that in most cases, while at first, the patient may need as much as ℥I to℥ISS of INTERNOL per day, with time, he can **diminish the dosage** to as little as half an ounce a day, or an ounce every other day, and even discontinue INTERNOL for periods of time.

In many cases, INTERNOL is the last resort to avoid another use of the surgeon's knife.

### THE INCREASE OF TISSUE RESISTANCE

The importance of increasing tissue resistance is best appreciated when one is called upon to treat a serious infection in a run-down person. Thus a pneumonia or bronchitis is so much more dangerous in an aged or debiliated person whose tissues have lost their usual power to throw off disease—that is, resist germ invasion.

For this purpose cod liver oil is of the utmost utility but an essential point is to choose a preparation that not only contains the active medicine and strengthening properties of the oil but which is also so palatable that its administration may be continued over long periods of time. Cord. Ext. Ol. Morrhuae Com. (Hagee) is such a product. A generation of doctors used it and learned to rely upon it.

Dr. R. M. Sterrett, many years associated with the advertising of Antiphlogistine, sends greetings to his many friends, announcing his resignation as Advertising Manager of the Denver Chemical Mfg. Co., effective, January 1st.

---

"I prescribe large quantities of Tongaline for many conditions for which it is particularly indicated, but I obtain especially gratifying results from its use for what are commonly called "colds," the symptoms of which are chills, fever, sneezing, coughing, etc., and for which the system requires, first of all, prompt and thorough elimination.

"I order 4 oucnes Tongaline, directing that two teaspoonfuls be taken in a half glass of water, preferably hot, every two hours for three or four doses or until the patient is thoroughly under the influence of the medicine; then one teaspoonful every three hours, until the attack has been aborted or the patint has recovered.

"I have personally derived great benefit from Tongaline for "colds," taking it on the first appearance of any of the symptoms and in nearly every instance abort the disease and I use several bottles of Tongaline myself during the winter months in that way."

### PROTECTION FOR OLD PEOPLE AGAINST BRONCHITIS.

With the onset of winter with its changeable weather, the old folks of reduced vitality and resistance, begin to suffer from bronchial inflammations. Prevention in these conditions is better than cure, and prevention lies in the employment of those agents that will add to the patient's bodily strength and more narrowly the resistance of bronchial tissue to atmospheric disturbances with a consequent germ invasion. For this purpose Cord. Ext. Ol. Morrhuae Comp. (Hagee) has been found of the utmost value, not alone for its therapeutic influence, but also by reason of the fact that it does not upset the stomach. Give it to your patients who suffer annually from bronchial attacks.

---

### INCREASES THE PHYSIOLOGIC EFFICIENCY OF THE STOMACH.

In no way is better shown the trend of modern medicine to revive and reinforce—rather than to supersede—the natural physiologic forces of the body, than in the present day treatment of functional gastric diseases. Only a short time ago there was one routine combination that was applied with almost religious fidelity in each and every case of stomach trouble, and that was pepsin and dilute hydrochloric acid· Fortunately an awakening came, and instead of supplying an artificial digestant, the practice to-day is to use measures that encourage the stomach to do its own work.

Among the therapeutic agents that have been found particularly effective in this respect, Seng easily stands at the forefront. Under its use the gastro-intestinal glands are stimulated, fermentation is promptly controlled, and the distress and discomfort that make the life of the average dyspeptic so miserable, rapidly disappear. And because these results are directly ude to the power of Seng to increase the physiologic efficiensy of the stomach, they are not temporary and fleeting; on the contrary, the benefits that Seng produces are prolonged, and with removal of causative conditions, the improvement obtained is as permanent as it is pronounced.

It is not difficult therefore to understand the place Seng has won in the regard of many a physician as the remedy of choice in all atonic forms of indigestion.

### WHEN THE HEART NEEDS HELP.

In every day practice there is a constant demand for a safe and relable cardiac tonic, a remedy that will correct irregularity of the heart's action, and at the same time give the aid and support needed to enable it to do its work satisfactorily.

Among the remedies that have been used to overcome the functional disorders of the heart, which, if not serious, nevertheless cause much anxiety to the afflicted patient, there is none thât has proven so generally useful as Cactina Pillets.

Non-cumulative in its effects, Cactina can be administered with every confidence in its power to support the tired, overworked heart, regulate its action and as one physician has expressed it, "help it over the hard places."

It is this dependable tonic action of Cactina that has led so many physicians to prefer it to other remedies in the treatment of tachycardia, tobacco heart, palpitation, arrhythmia and functional diseases of th eheart generally. Its efficiency is shown in no uncertain way by the aid afforded "when the heart needs help."

---

### DESIDERATA OF AN ANODYNE

In making a choice of an anodyne the careful physician demands these properties of such an agent, viz., that it be therapentically active in moderate dosage, that it be practically safe, especialy with women and children, and that it produce a minimum of evil effects. Since PAPINE (Battle) easily meets these requiremnts of the exacting clinician. It has come into wide favor, and, in fact, is enjoying an ever increasing use among discriminating physicians.

---

**REPRINTS**

Of Original Articles published in the Journal-Record of Medicine will be furnished at the following prices:

500 Copies 50c per page.

1000 Copies 75c per page.

When Reprints are desired it should be indicated on the copy.

**SAL HEPATICA**

AN EFFERVESCING

SALINE COMBINATION

LAXATIVE AND

ELIMINANT

BRISTOL-MYERS CO.

NEW YORK

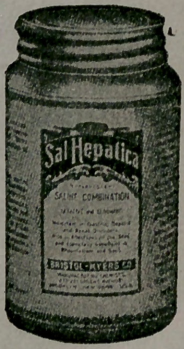

### Starch and Table Salt Sold as Neosalvarsan.

The recent indictment by the Federal Grand Jury in New Ark, N. J., of "Dr." Jean F. Strandgaard, of Toronto, Canada, and George F. Hardacre, of Toronto, and a steward on the steamship "United States," has revealed to Chief Inspector E. R. Norwood, of the Customs Service in New York, what he believes to be a widespread conspiracy to defraud the Government out of customs revenue by smuggling salvarsan and neosalvarsan into the United States.

A most serious feature of this matter is the discovery by Inspector Norwood that these men also had in their possession a large quantity of spurious neosalvarsan. Upon analysis by the Government experts, the contents proved to be starch in the majority of the ampules and stained table salt in the others.

A further investigation showed that during July, 1916, Strandgaard had 15,000 ampules made in Jersey City, which upon his instructions were filled by the glass blower with either starch or salt. A remarkable coincidence is that during August and September, and as recently as the time Strandgaard was arrestetd in New York, physicians and drug stores all over the Middle West and the East were approached by women trying to sell, on the one pretense or another, the frauds made for Strandgaard. These spurious products were put up in imitation of either the German or particularly the English package, as marketed by the German manufacturers in England before the war, in square pasteboard cartons. They did not appear in round aluminum packages, like the American package. They are very cleverly executed, and their outside appearance even led experienced physicians to be deceived.

The product has been sold in New York, Chicago, Milwaukee, Cincinnatti, Peoria, Kalamazoo, Detroit, Terre Haute, and Mobile, and other Western and Southern cities, and is undoubtedly still being peddled on account of the great profits accruing to the saleswomen.

There is no need to call the attention of physicians to the dangers connected with the use of such frauds. In view of the serious and possible fatal results which would follow the administration of these fraudulent salvarsans, it is incumbent upon medical men who have any information about the distribution or sale of these frauds to communicate with Chief Inspector E. R. Norwood, U. S. Customs House, New York, at their earliest opportunity, or, in case of emergency, with the local police authorities.

